# An Identity-Based Anti-Quantum Privacy-Preserving Blind Authentication in Wireless Sensor Networks

**DOI:** 10.3390/s18051663

**Published:** 2018-05-22

**Authors:** Hongfei Zhu, Yu-an Tan, Liehuang Zhu, Xianmin Wang, Quanxin Zhang, Yuanzhang Li

**Affiliations:** 1 School of Computer Science and Technology, Beijing Institute of Technology, Beijing 100081, China; farseer@bit.edu.cn (H.Z.); tan2008@bit.edu.cn (Y.T.); liehuangz@bit.edu.cn (L.Z.); zhangqx@bit.edu.cn (Q.Z.); 2 School of Computer Science, Guangzhou University, Guangzhou 510006, China; xianmin@gzhu.edu.cn

**Keywords:** identity-based blind signature, quantum computer attack, NTRU lattice, unforgeability

## Abstract

With the development of wireless sensor networks, IoT devices are crucial for the Smart City; these devices change people’s lives such as e-payment and e-voting systems. However, in these two systems, the state-of-art authentication protocols based on traditional number theory cannot defeat a quantum computer attack. In order to protect user privacy and guarantee trustworthy of big data, we propose a new identity-based blind signature scheme based on number theorem research unit lattice, this scheme mainly uses a rejection sampling theorem instead of constructing a trapdoor. Meanwhile, this scheme does not depend on complex public key infrastructure and can resist quantum computer attack. Then we design an e-payment protocol using the proposed scheme. Furthermore, we prove our scheme is secure in the random oracle, and satisfies confidentiality, integrity, and non-repudiation. Finally, we demonstrate that the proposed scheme outperforms the other traditional existing identity-based blind signature schemes in signing speed and verification speed, outperforms the other lattice-based blind signature in signing speed, verification speed, and signing secret key size.

## 1. Introduction

With the development of wireless sensor networks, Internet of Things (IoT) devices play an important role in smart cities. IoT devices in e-payment and e-voting services are crucial for modernisation [1,2,3]. Meanwhile, a large amount data generated by these IoT devices face the threats of security and privacy leakage since the state-of-art authentication protocols in e-payment and e-voting systems can be attacked by quantum computers successfully [4], i.e., in e-payment and e-voting systems, blind signature (BS) is crucial to protect user privacy and guarantee trustworthy of big data in the cloud  [5,6,7,8]. However, these schemes based on traditional number theory can be attacked successfully by quantum computer.

BS was firstly introduced by Chaum. Then many BS schemes based on number theory were proposed [9], which can be presented as follows:

The first factoring BS scheme based on RSA was proposed by Chaum, this scheme can guarantee the security of payer. However, they did not prove its security. Later, Bellare et al. defined the hard problem of RSA formally. Based on it, they proved the security of Chaum’s scheme. Then a novel proven-secure RSA scheme was proposed by Camenisch and Koprowski etc., it was secure in the standard model. However, these schemes have to use long keys to guarantee security.

In order to overcome the shortages of factoring BS schemes, BS schemes based on discrete logarithm problem (DLP) were proposed for their short keys and high security. Chaum et al. proposed an e-wallet. Later, Okamoto proposed a BS scheme based on DLP. However, these schemes were not proven secure and only satisfy blindness. Then Pointcheval et al. initially considered the property of unforgeability.

After that, researchers were interested in constructing provably-secure BS schemes based on bilinear pairing. Boldyreva proposed a BS scheme based on GDH assumption, this scheme outperformed the other existing schemes in attribution and efficiency. Later, Okamoto proposed a BS scheme based on 2SDH assumption, which is stronger than SDH assumption. However, their efficiency is low.

Meanwhile, all the schemes outlined above need to depend on Public Key Infrastructure (PKI). In order to simplify key management of PKI, an identity-based signature scheme (IDS) was firstly presented by Shamir. In an IDS scheme, given a user’s identity, his public key can be easily obtained. Also, his private key can be obtained easily. Until 2001, Boneh et al. initially proposed an IDS scheme, it has high efficiency, its security is dependent on the bilinear pairing problem. Then some new IDS schemes based on pairing were proposed by researchers. After that, combining BS with identity-based signature, Zhang et al. initially presented an identity-based BS (IDBS) scheme, its security is based on hard problem of bilinear pairing, this scheme was secure and efficient. Unfortunately, its computation cost was too high. Later, a new IDBS based on DLP was presented, the running time and signature size of their scheme [10] were significantly improved. However, these schemes still face the threat of quantum computer attack [4].

Thus, the replaceable IDBS schemes are based on lattice for their high-efficiency and sufficiently secure to quantum computer attack [11,12]. In the paper, a lattice-based IDBS scheme is proposed by using the advantages of number theory research unit lattice (NTRU) such as high efficiency, extremely tight keys, and sufficient safety once properly parameterized.

(1) Inspired by [13,14,15], we propose a new IDBS scheme on NTRU Lattice (named IDBS-NTRU), which can be secure to resist quantum computer attack.
(2) We evaluate our IDBS-NTRU’s security. We demonstrate that the proposed scheme is secure. Then we prove that the proposed scheme satisfies confidentiality, integrity, and non-repudiation.
(3) We compare our IDBS-NTRU’s performance with the other IDBS schemes.
  - Comparing with existing traditional IDBS schemes, its signing speed is faster than other schemes, its moves are shorter than other schemes, its signing secret key, and signature size are larger than other schemes.
  - Comparing with existing lattice-based BS schemes, its signing speed is faster than other lattice-based BS schemes, its moves are shorter than Rückert and ZM schemes, its signing secret key is smaller than other lattice-based schemes, and its signature length is smaller than Rückert scheme.

Organization. Section 2 presents the definitions of NTRU lattice and IDBS. Section 3 shows how to design an IDBS scheme. Section 4 proves the proposed IDBS’s security, and compares with the existing IDBS schemes in terms of performance. Lastly, we conclude the paper in Section 5.

## 2. Preliminaries

### 2.1. The Applications for BS

**Figure 1:**
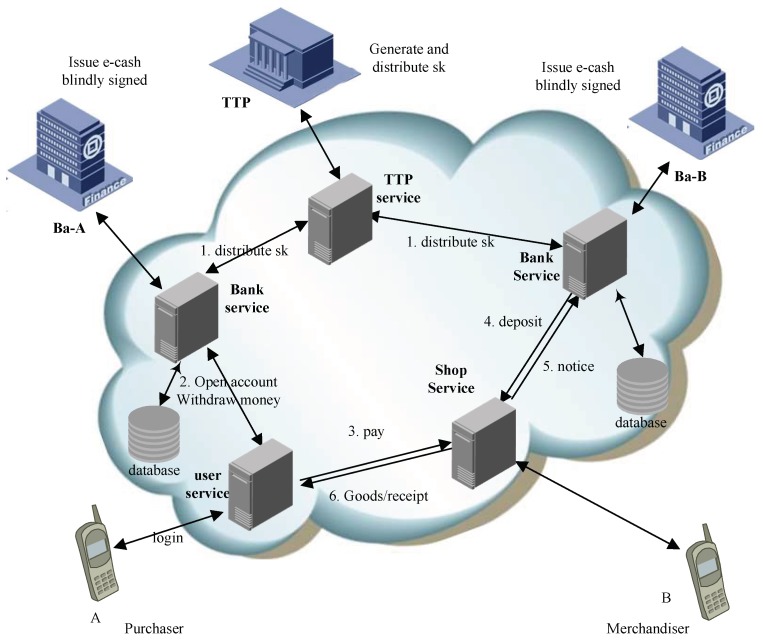
Blind authentication in e-payment system.

With the development of big data, which has the properties of volume, variety, velocity, value, veracity, variability, viscosity, and virality, organizations deploy their services such as e-payment and e-voting systems etc. to the cloud [16,17,18]. In e-payment and e-voting systems, BS scheme plays an important role for that BS scheme can protect user’s anonymous instead of encrypting all the data and searching on the ciphertexts [19,20,21]. In addition, scholars proposed some methods to protect security in the cloud [22,23,24,25], which can provide us with new methods to make our scheme in practice. Meanwhile, scholars proposed some methods to detect complex event analysis, which can be used to improve the security of these services and applications in the cloud [26,27]. We will briefly describe e-payment and e-voting systems as follows:

E-payment system: *A*, *B*, *T*, and Ba are denoted as buyer, merchandiser, trusted third party, and bank respectively. Then the e-payment process is presented in Figure 1 [4]. In the beginning, *T* will produce and deliver keys for all the Bas, A,B will open a new account from their Ba respectively. The details are as follows:

*A* logins into his account, draws e-cash *m* from the Ba-*A*, blinds *m* by using blind factor *f*, and then obtains $m^{{}^{\prime }}$. The Ba-*A* signs on $m^{\prime }$, and sends the signature $\sigma^{\prime }$ to *A* [28]. *A* unblinds the signature by using *f* and obtains σ. *A* sends the tuple <m,σ> to *B*. *B* verifies whether it is valid or not, if it is, he sends the tuple to Ba-*B*. The Ba-*B* deposits the money on *B*’s account.

**Figure 2:**
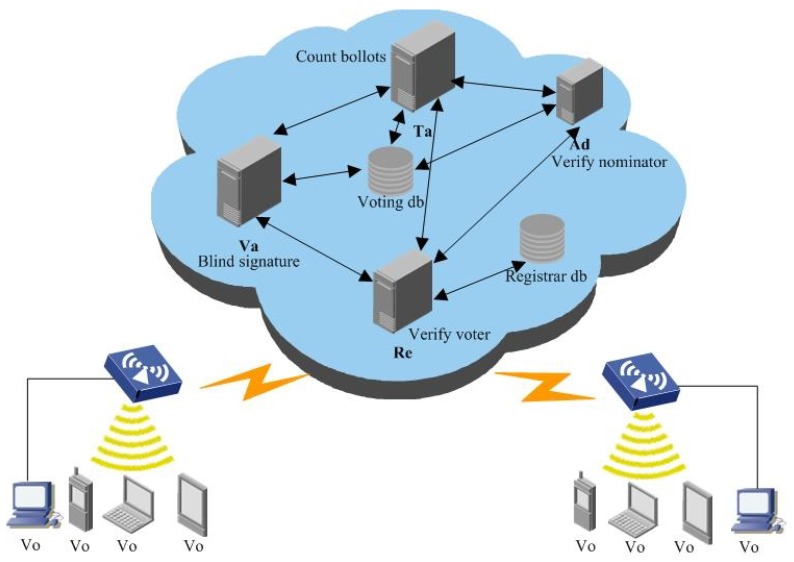
Blind authentication in e-voting system.

E-voting system: the voter, registrar, administrator, tallier, nominators, and validator are denoted as vo, re, ad, ta, no, and va respectively. The protocol is presented in Figure 2 [4]:

vo sends his id to a re, the re checks whether the vo is valid. If he is, the vo can send two nos to ad, the ad will check whether they are valid. If they are, the vo can choose a ballot *m*, blind it by using blind factor *f*, and then get the blinded message $m^{\prime }$. $m^{\prime }$ will be sent to a va, the va signs on it and sends the signature $\sigma^{\prime }$ to the vo. The vo unblinds $\sigma^{\prime }$ by using blind factor *f*, and gets a signature σ. The vo sends m,σ to a ta, the ta will count all his ballots and store the results to a voting database.

### 2.2. NTRU Lattice, Gaussians Sampling and Rejection Sampling on Lattice

Let α and γ be the vectors, *p* and $N=2^{p}$ be integers, *q* be a prime which is greater than 5. Then we denote $R=Z\left[x\right]/\left(x^{N}+1\right)$ as a ring. We denote $f=\Sigma_{i=0}^{N-1}f_{i}x^{i}$ and $g=\Sigma_{i=0}^{N-1}g_{i}x^{i}$ as polynomials in *R*. $R^{\times }$ is a set that all the elements have inverse in *R*. We write <α,γ> as vectors’ inner product and ||α|| as α’s Euclidean norm. We write $R_{q}=Z_{q}\left[x\right]/\left(x^{N}+1\right)$ as the ring. We denote polynomial multiplication and concatenation as f,g mod $\left(x^{N}+1\right)$ and $\left(f,g\right)\in R^{2N}=R^{1\times 2}$ in *R* respectively.

Next, we introduce the definitions of NTRU lattice, Gaussians sampling [29], and Rejection sampling [14]. NTRU lattice is used for constructing NTRUEncrypt and NTRUSign. These cryptosystems have high-efficiency, extremely tight keys, and are sufficiently secure once properly parameterized. The NTRU lattice is introduced as follows:

**Definition** **1**  (NTRU lattice)**.** *Let d,e∈R, $h=e\times d^{-1}$ mod q. Then $L_{h,q}=\{u,v\in R^{2}:u+v\times h=0$ mod q} is defined as NTRU Lattice. Meanwhile, $L_{h,q}$ is a $R^{2N}$ full-rank lattice $\left(\begin{array}{cc} -C(h) & I\\ qI & O \end{array}\right)$ , in which I is a unit matrix, O is a null matrix, C(h) is a matrix as follows: $\left(\begin{array}{ccc} h_{0} & h_{1}\ldots & h_{N-1}\\ -h_{N-1} & h_{0} & h_{N-2}\\ . & . & .\\ -h_{1} & -h_{2} & h_{0} \end{array}\right)$.*

The security of our IDBS is based on R-SIS problem over NTRU lattice, it is defined as follows:

**Definition** **2**  ($R-SIS{}_{q,1,2,\beta }^{\kappa }$ on NTRU lattice)**.** *in a ring $R=Z\left[x\right]/\left(x^{N}+1\right)$, κ is denoted as a distribution, in which we can choose small f,g from $D_{Z^{N},\sigma }$ (f,g mod $q\in R_{q}^{\times }$) according to the Algorithm 3 in [13], then we can get $B_{h,q}=\left(h,1\right)\in R_{q}^{1\times 2}$, $h=gf^{-1}$. Thus, the SIS problem means to search $\zeta_{1},\zeta_{2}$ meeting $B_{h,q}{\left(\zeta_{1},\zeta_{2}\right)}^{T}=0$ mod q, and $||(\zeta_{1},\zeta_{2}||\leq \beta$.*

Gaussian sampling was used for constructing the trapdoor in [29], i.e., a short basis was used to construct the trapdoor without revealing anything about this basis.

**Definition** **3**  (Discrete Gaussian Distribution)**.** *for ∀s>0, $x\in R^{N}$, and the center of Gaussian distribution c, the N-dimensional Gaussian function can be defined as $\rho_{s,c}\left(x\right)=exp\left(\frac{-\pi ||x-{c||}^{2}}{s^{2}}\right)$. Then the discrete Gaussian distribution on L can be defined as $D_{L,s,c}\left(x\right)=\frac{\rho_{s,c}\left(x\right)}{\rho_{s,c}\left(L\right)}$.*

Given real ψ>0, negligible probability ψ(n), a lattice L, and its smoothing parameter $\eta_{\epsilon }\left(L\right)\leq \sqrt{log(2N/(1+1/\epsilon ))/\Pi }/\lambda_{1}^{\infty }\left(L^{*}\right)$, there always exists ψ(n) for $\eta_{\epsilon }\left(L\right)\leq \omega \left(\sqrt{logN}\right)/\lambda_{1}^{\infty }\left(L^{*}\right)$ given any $\omega (\sqrt{logN})$ function. If $s>\eta_{\epsilon }\left(L\right)$, then the total Gaussian measure on all the kinds of translation of the lattice is the same according to Lemma 2.7 in [29]. If $s>2\eta_{\epsilon }\left(L\right)$, then $D_{L,s,c}\left(x\right)\leq \left(1+\epsilon \right)2^{-N}/\left(1-\epsilon \right)$. If $\epsilon <\frac{1}{3}$, then the min-entropy of $D_{L,s,c}\left(x\right)$ is at least N−1 according to Lemma 2.10 in [29].

**Lemma** **1.** *The two events occur with probability $pr[y\leftarrow D_{\sigma }^{1}:||y||\geq 12\sigma ]<2^{-100}$ (σ>0), $pr[y\leftarrow D_{\sigma }^{m}:||y||\geq 2\sigma \sqrt{m}]<2^{-m}$ (m is a non-negative integer) according to Lemma 3.3 in [14]. Let B be a basis of L, σ,c be the standard deviation and the center of Gaussian distribution respectively. We can get the desired vectors from the discrete Gaussian sampling algorithm in Algorithm  1.*

**Algorithm 1**Gausssian(B,σ,c).
1:Input: B, σ>0, *c*2:Output: v3:$v_{n}\leftarrow 0$ and $c_{n}\leftarrow c$.4:for(i←N to 1)5: (a) $c_{i}^{{}^{\prime }}=<c_{i},\tilde{b_{i}}>/\left|\right|\tilde{b_{i}}{\left|\right|}^{2}$6: (b) choose $z_{i}∼D_{Z^{N},s_{i}^{{}^{\prime }},c_{i}^{{}^{\prime }}}$7: (c) $c_{i-1}\leftarrow c_{i}-z_{i}b_{i}$ and $v_{i-1}\leftarrow v_{i}+z_{i}b_{i}$8: end for9:return $v_{0}$


Next, we begin to introduce the Rejection-sampling. In a signature scheme, rejection sampling can make the output signature distribution not depend on the signing key.

**Theorem** **1.** *[Rejection Sampling Theorem] V is the subset of $Z^{m}$, the norms of V’s elements are less than T, $\sigma =\omega (T\sqrt{logm})$ is the element in R, M is a constant, h:V→R is a probability distribution. There are two algorithms. One algorithm is such that $x\leftarrow h,y\leftarrow D_{v,\sigma }^{m},outputs\left(x,y\right)$ with probability $min(\frac{D_{\sigma }^{m}\left(y\right)}{MD_{v,\sigma }^{m}\left(y\right)},1)$. The other algorithm is such that $x\leftarrow h,y\leftarrow D_{\sigma }^{m},outputs\left(x,y\right)$ with probability $\frac{1}{M}$. Then the first algorithm’ distribution does not exceed the second algorithm’s statistical distance $\frac{2^{-\omega (logm)}}{M}$. Meanwhile, the first algorithm outputs something with probability at least $\frac{1-2^{-\omega (logm)}}{M}$.*

In particular, when σ=αT,α is positive, then $M=e^{\frac{12}{\alpha }+\frac{1}{2\alpha^{2}}}$, the first algorithm’s distribution does not exceed the second algorithm’ statistical distance $\frac{2^{-100}}{M}$. The first algorithm outputs something with probability at least $\frac{1-2^{-100}}{M}$.

### 2.3. IDBS

An IDBS scheme consists of four algorithms $\left(ST_{\varepsilon },EX_{\varepsilon },SG_{\varepsilon },VF_{\varepsilon }\right)$, U, S, and V are denoted as user, signer, and verifier respectively. Master key, master public key, and master private key are severally written as mk, mpk, and msk. System parameters are denoted as params, *n* is the security parameter. The definition is described as follows.

- $ST_{\varepsilon }\left(1^{n}\right)$: after inputting *n*, this algorithm outputs params and mk, which contains mpk and msk.
- $EX_{\varepsilon }\left(params,msk,id\right)$: after inputting params, msk, id, this algorithm outputs private key $sk_{id}$ related to id.
- $SG_{\varepsilon }\left(id,m,sk_{id}\right)$: U interacts with S as follows:
  (1) U blinds the message *m* to $m^{{}^{\prime }}$ by using blind factor, then sends $m^{{}^{\prime }}$ to S.
  (2) S signs on $m^{{}^{\prime }}$ and sends the signature $\sigma^{{}^{\prime }}$ to U.
  (3) U unblinds $\sigma^{{}^{\prime }}$ and gets σ. The signature tuple is (m,σ).
- $VF_{\varepsilon }\left(params,id,m,\sigma \right)$: this algorithm returns true if σ is valid, otherwise returns false.

Before introducing the security properties of IDBS, we define some notations firstly. Γ is denoted as an adversary, *U* is nonmalicious users, *m* is the plaintext message, c,n are denoted as a constant and a big integer respectively, η is a negligible probability, *t* is the time.

IDBS should achieve two properties, which are defined as follows [30,31]:

***Blindness*** [32]: Γ chooses two messages $m_{0},m_{1}$, then a random bit *i* is selected, $m_{0},m_{1}$ are randomly denoted as $m_{i},m_{1-i}$, $m_{i},m_{1-i}$ are the inputs of two honest users respectively. Γ plays the Experiment 1 with these two users, $\sigma_{i},\sigma_{1-i}$ are the outputs of them respectively. $\sigma_{i},\sigma_{1-i}$ are dispatched to Γ, after that, Γ will output a bit p∈{0,1}. Finally, the probability of p=i is denoted as |Pr[p=i]−1/2|<η(n). i.e., if no Γ can win the Experiment 1 at the minimum with η in *t*, then it satisfies blindness.

***One-more unforgeability***  [4]: after Γ interacts with a nonmalicious signer for *l* times, he tries to forge the l+1 valid signature with η. The game is defined in Experiment 2. i.e., if Γ cannot win the Experiment 2 with η at most $\tau_{1},\tau_{2},\tau_{3}$ times respectively for extraction, hash, and signature oracles in *t*, then the scheme satisfies one-more unforgeability.

**Experiment 1**$Expt_{S^{*}}^{bd}\left(n\right)$.

$i\in_{\$}\left\{0,1\right\}$

$\left(params,msk\right)\leftarrow ST\left(1^{n}\right)$

$sk_{id}\leftarrow EX\left(params,id,msk\right)$

$\left(m_{0},m_{1},state_{find}\right)\leftarrow_{\$}S^{*}\left(find,sk_{id},id\right)$

$state_{issue}\leftarrow_{\$}S^{*<.,U{\left(id,m_{i}\right)}^{1}>,<.,U{\left(id,m_{1-i}\right)}^{1}>}\left(issue,state_{find}\right)$
$\delta_{i}$, $\delta_{1-i}$ are respectively $U\left(id,m_{i}\right),U\left(id,m_{1-i}\right)$’s outputs**if**$\delta_{0}\neq fail$ and $\delta_{1}\neq fail$ **then** $p\leftarrow_{\$}S^{*}\left(guess,\delta_{0},\delta_{1},state_{issue}\right)$

**else**
 $p\leftarrow_{\$}S^{*}\left(guess,fail,fail,state_{issue}\right)$

**end if**
return true iff p=i


**Experiment 2**$Expt_{U^{*}}^{omf}\left(n\right)$.

$\left(params,msk\right)\leftarrow ST\left(1^{n}\right)$

$sk_{id}\leftarrow EX\left(params,id,msk\right)$

$\left\{\left(m_{1},s_{1}\right),...,\left(m_{k},s_{k}\right)\right\}\leftarrow_{\$}U^{*h\left(.\right),<S\left(sk_{id}\right),.{>}^{\infty }}\left(id\right)$
*l* is the successful interaction number between $U^{*}$ and signerreturn true iff   $m_{i}\neq m_{j}$ for 1≤i<j≤k and   $VF(m_{i},s_{i},id)=1$ and   l+1=k


## 3. Proposed IDBS-NTRU Scheme

Most IDBS schemes are designed with the traditional number theorem; these schemes cannot defeat a quantum computers attack. So the replaceable IDBS schemes are based on lattice. Meanwhile, NTRU-cryptosystems have some advantages, such as high-efficiency, extremely tight keys, and sufficient safety after properly parameterized. Therefore, we choose the NTRU lattice to construct a novel IDBS scheme so that we can achieve both security and efficiency.

In this section, we will firstly introduce how to construct an IDBS scheme on NTRU lattice, then we design an e-payment protocol using our proposed scheme.

### 3.1. IDBS-NTRU Scheme

**Figure 3:**
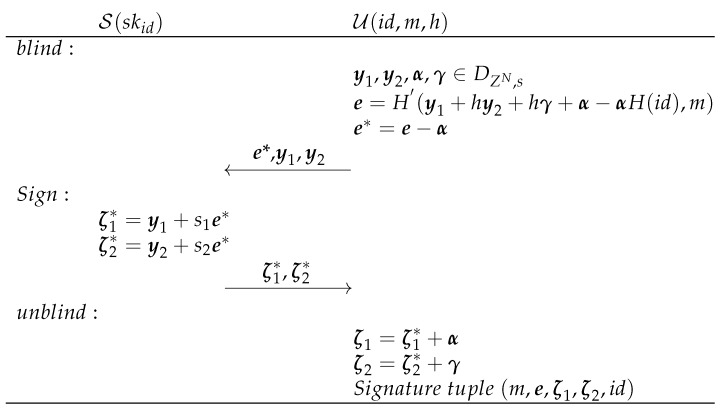
Proposed IDBS-NTRU protocol.

In this section, we propose our IDBS scheme $\varepsilon =(ST_{\varepsilon },EX_{\varepsilon },SG_{\varepsilon },$$VF_{\varepsilon })$. Let U, S, V be a user, a signer, and a verifier respectively, *N* and id be security parameter and user’s identity respectively, $\tilde{\Omega }\left(.\right)$ and Poly(N) be the asymptotic lower bound and *N*’s polynomial function respectively [13].

(1) $ST_{\varepsilon }\left(1^{N}\right)$ outputs (params=(q,ε,s),mk=(sk,pk)), in which q=Poly(N), $\varepsilon \in (0,\frac{\ln N}{\ln q})$, and $s=\tilde{\Omega }\left(N^{\frac{3}{2}}\sigma \right)$. If N>2, then $\sigma =N\sqrt{\left(\ln(8Nq\right)}q^{\frac{1}{2}+\varepsilon }$, $q^{1/2-\varepsilon }=\tilde{\Omega }\left(n^{\frac{7}{2}}\right)$. If N=2, then $\sigma =\sqrt{N\ln(8Nq)}q^{\frac{1}{2}+\varepsilon }$, $q^{\frac{1}{2}-\varepsilon }=\tilde{\Omega }\left(N^{3}\right)$. mk is generated as follows [13]:
  The algorithm samples f,g from $D_{Z^{N},s}$, which satisfy f,g mod $q\notin R_{q}^{\times }$. Meanwhile, $\left||f||,||g|\right|\leq \sigma \sqrt{N}$ and <f,g>∈R. Then the algorithm computes $F_{1},G_{1}\in R$, which satisfy $fG_{1}-gF_{1}=1$. We compute $F_{q}=qF_{1},G_{q}=qG_{1}$, and then obtain (F,G) by using babai algorithm in [11], which satisfies $\left(F,G\right)=\left(F_{q},G_{q}\right)-k\left(f,g\right)$, k∈R. If ||(F,G)||≤Nσ, then outputs sk = D=$\left(\begin{array}{cc} C(f) & C(g)\\ C(F) & C(G) \end{array}\right)$ and $pk=h=gf^{-1}\in R_{q}^{\times }$.
(2) $EX_{\varepsilon }\left(params,id,sk\right)$ computes t←H(id), and $sk_{id}=\left(s_{1},s_{2}\right)\leftarrow \left[(t,0\right)$−Gausssian(sk,σ,(t,0))], in which $s_{1}+s_{2}*h=t$. Then the algorithm outputs $sk_{id}$ to user id [13].
(3) $SG_{\varepsilon }$: Let $m\in {\left\{0,1\right\}}^{*}$ be the plaintext, U randomly selects $y_{1},y_{2},\alpha ,\gamma \in D_{Z^{N},s}$, then U executes BS protocol in Figure 3.
  - U computes
    (1) $$\begin{array}{c} e=H^{{}^{\prime }}\left(y_{1}+hy_{2}+h\gamma +\alpha -\alpha H\left(id\right)\right),m) \end{array}$$
    and
    (2) $$\begin{array}{c} e^{*}=e-\alpha \end{array}$$
    then U sends $e^{*}$ to S.
  - S computes Equations (3) and (4), then sends $\zeta_{1}^{*},\zeta_{2}^{*}$ to U.
    (3) $$\begin{array}{c} \zeta_{1}^{*}=y_{1}+s_{1}e^{*} \end{array}$$
    (4) $$\begin{array}{c} \zeta_{2}^{*}=y_{2}+s_{2}e^{*} \end{array}$$
    Here, we will explain how to use the rejection sampling theorem, Theorem 1 from Section 2.2. The core idea of this theorem is to make $\zeta_{1},\zeta_{2},e^{*}$ do not rely on the private key $s_{1},s_{2}$ respectively. Our target is that the distribution of $\zeta_{1},\zeta_{2}$ will obey the distribution $D_{\sigma }^{N}$. However, $\zeta_{1},\zeta_{2}$ obey the distribution $D_{v,\sigma }^{N}$, where $c=v_{1}$ or $v_{2}$, $v_{1}=s_{1}e^{*}$, and $v_{2}=s_{2}e^{*}$. After we appropriately choose a certain *M* and σ, the algorithm will approximately output a signature tuple with probability 1/M, whose distribution is approximate to the distribution where $\zeta_{1},\zeta_{2}$ are chosen from $D_{\sigma }^{N}$ [14].
  - Finally, U gets the signature tuple <$m,\zeta_{1},\zeta_{2},e,id$> from Equations (5) and (6) with probability $min(\frac{D_{Z^{N},s}}{MD_{Z^{N},s,sk_{id}e^{*}}},1)$, in which *M* is a constant.
    (5) $$\begin{array}{c} \zeta_{1}=\zeta_{1}^{*}+\alpha \end{array}$$
    (6) $$\begin{array}{c} \zeta_{2}=\zeta_{2}^{*}+\gamma \end{array}$$
(4) $VF_{\varepsilon }\left(m,e,\zeta_{1},\zeta_{2},id\right)$: V validates whether Equations (7) and (8) is true. If it is, accept it. Otherwise reject it.
  (7) $$\begin{array}{c} \left|\right|\left(\zeta_{1},\zeta_{2}\right)\left|\right|\leq 4s\sqrt{2N} \end{array}$$
  (8) $$\begin{array}{c} H^{{}^{\prime }}\left(h*\zeta_{2}+\zeta_{1}-H\left(id\right)*e,m\right)=e \end{array}$$

### 3.2. An E-Payment Protocol

**Figure 4:**
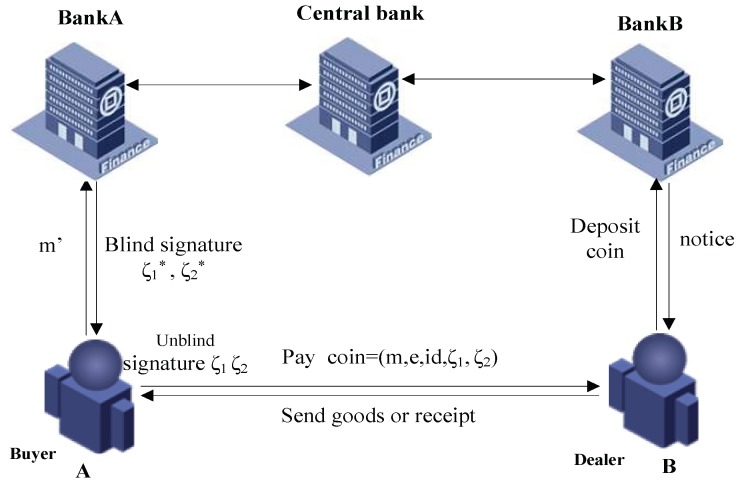
A buys goods from B.

In this section, we design an e-payment protocol based on NTRU-IDBS scheme, which plays an important role in e-commerce. We will still follow the notations in Section 2.1. As described in Figure 4, *A*’s account belongs to bankA, *B*’s account belongs to BankB. Firstly, *A* draws e-money from BankA. Secondly, *A* pays the money to *B*. Finally, *B* deposits the money to BankB. Following is the details:

(1) *T* produces and sends keys
  - *T* runs the algorithm $ST_{\epsilon }$ and produces the system parameter params and master key mk.
  - *T* runs algorithm $EX_{\epsilon }$ and generates the keys for BankA and BankB.
  - BankA’s public key and private key are $id_{BankA},sk_{BankA}$ respectively.
  - BankB’s public key and private key are $id_{BankB},sk_{BankB}$ respectively.
  - *T* distributes the corresponding private keys to BankA and BankB.
(2) user opens an account from Bank
  - *A* and *B* open an account using their real identity, such as passport, ssn, address, email, male, age, and so on, their banks will give them their account information respectively.
(3) *A* draws e-money from BankA
  - *A* send their account information to BankA.
  - BankA will verify whether he is a valid user. If it is, continue. Otherwise, abort.
  - *A* wants to draw money *m*, he will randomly choose vectors $y_{1},y_{2},\alpha ,\gamma$, computes $e=H^{{}^{\prime }}\left(y_{1}+hy_{2}+h\gamma +\alpha -\alpha H\left(id\right),m\right)$ and $e^{*}=e-\alpha$ to obtain $e^{*}$.
  - *A* sends *m* with the blinded note $e^{*}$ to BankA.
  - BankA computes $\zeta_{1}^{*}=y_{1}+s_{1}e^{*}$, $\zeta_{2}^{*}=y_{2}+s_{2}e^{*}$ for $e^{*}$, and generates the signatures $\zeta_{1}^{*}$ and $\zeta_{2}^{*}$, then records on the account of *A*.
  - Next, the bank returns $\zeta_{1}^{*},\zeta_{2}^{*}$ to *A*.
  - *A* computes $\zeta_{1}=\zeta_{1}^{*}+\alpha$ and $\zeta_{2}=\zeta_{2}^{*}+\gamma$ to get $\zeta_{1},\zeta_{2}$.
(4) *A* pays the e-money to *B*
  - *A* sends $m,e,\zeta_{1},\zeta_{2},id$ to *B*.
  - *B* computes $\left|\right|\left(\zeta_{1},\zeta_{2}\right)\left|\right|\leq 4s\sqrt{2N}$, $H^{{}^{\prime }}\left(h*\zeta_{2}+\zeta_{1}-H\left(id\right)*e,m\right)=e$ and checks whether all of them are true. If all are true, accept it, otherwise, reject them.
(5) *B* deposits the e-money
  - *B* will send $m,e,\zeta_{1},\zeta_{2},id$ to BankB.
  - BankB computes and checks whether $\left|\right|\left(\zeta_{1},\zeta_{2}\right)\left|\right|\leq 4s\sqrt{2N}$, and $H^{{}^{\prime }}\left(h*\zeta_{2}+\zeta_{1}-H\left(id\right)e,m\right)=e$ are true, if all of them are true, continue; otherwise abort.
  - BankB checks whether the e-money is in the list. If it is, abort, otherwise, continue.
  - BankB will deposit the e-money on B’s account.
  - BankB will send a notice to *B* that *B* has received the e-money.
  - *B* will send the goods or receipt to *A*.

## 4. Analyzing the Security and Performances

Here, we evaluate our IDBS-NTRU scheme with regard to correctness and security, then we compare the IDBS-NTRU scheme with other IDBS schemes in terms of performance.

### 4.1. Correctness, Blindness and One-More Unforgeability

**Theorem** **2**  (Correctness)**.** *The IDBS-NTRU scheme is correct.*

**Proof.**  Following our IDBS-NTRU scheme, we can get

(9) $$\begin{array}{cc} h & *\zeta_{2}+\zeta_{1}-H\left(id\right)*e\\ & =h\left(\zeta_{2}^{*}+\gamma \right)+\zeta_{1}^{*}+\alpha -H\left(id\right)*e\\ & =h\left(y_{2}+s_{2}e^{*}+\gamma \right)+y_{1}+\alpha +s_{1}e^{*}-H\left(id\right)*e\\ & =y_{1}+hy_{2}+h\gamma +\alpha -\alpha H\left(id\right) \end{array}$$

Thus, $H^{{}^{\prime }}\left(h*\zeta_{2}+\zeta_{1}-H\left(id\right)*e,m\right)=e$.

By using Lemmas 2 and 3 in [13], the distributions of $\zeta_{1}^{*},\zeta_{2}^{*}$ are close to $D_{Z^{N},s}$, α,γ are the vectors from $D_{Z^{N},s}$. So the probability of $\left|\right|\zeta_{1}\left||,|\right|\zeta_{2}\left|\right|\leq 4s\sqrt{N}$ is at least $1-2^{-N}$. Then we can get $\left|\right|\left(\zeta_{1},\zeta_{2}\right)\left|\right|\leq 4s\sqrt{2N}$.

To prove IDBS-NTRU scheme’s blindness, we introduce the statistical distance theorem, that is crucial to prove blindness property. ☐

**Theorem** **3**  (Statistical Distance Theorem)**.** *let random variable number P,Q∈Ω, in which *Ω* is a finite domain. The statistical distance equation is presented as below [33]:*

(10) $$\begin{array}{c} \Delta \left(P,Q\right)=1/2\sum_{\omega \in \Omega }\left|Pr\left[P=\omega \right]-Pr\left[Q=\omega \right]\right| \end{array}$$

When we prove IDBS-NTRU’s blindness, the malicious $S^{*}$ will play the Experiment 1 with two trust users respectively.

**Theorem** **4**  (Blindness)**.** *The IDBS-NTRU satisfies blindness.*

**Proof.**  A random bit i←{0,1} is chosen, which is kept secret from $S^{*}$. Then $S^{*}$ chooses $m_{0},m_{1}$, then $S^{*}$ interacts with two honest users as in Experiment 1. Following is the protocol:

- $\left(pk,sk\right)\leftarrow KG_{\varepsilon }\left(1^{k}\right)$
- $sk_{id}\leftarrow EX\left(params,id,sk\right)$
- Under finding mode, $S^{*}$ selects $m_{0},m_{1}\leftarrow S^{*}\left(1^{k},id,sk_{id}\right)$.
- Under issuing mode, a random bit *i* is selected randomly, that cannot be obtained by $S^{*}$. Then $m_{0},m_{1}$ are randomly denoted as $m_{i},m_{1-i}$ respectively. $S^{*}$ concurrently interacts with $U(id,m_{i})$ and $U(id,m_{1-i})$ .
- If one user outputs $\delta (m_{i})$, the other outputs $\delta (m_{1-i})$, we will send a sequence < $\delta \left(m_{i}\right),\delta \left(m_{1-i}\right)$> to $S^{*}$.
- Under guessing mode, $S^{*}$ returns $\tilde{i}$.

As in Figure 3, the Interactive values do not depend on *m*, so what we need to do is analyzing $e^{*},y_{1},y_{2},\zeta_{1}^{*},\zeta_{2}^{*}$.

For $e^{*}$, the statistical-distance is defined as follows

(11) $$\begin{array}{c} \Delta \left(e_{i}^{*},e_{1-i}^{*}\right)=1/2\sum_{e^{{*}^{\prime }}\in D_{Z^{N},s}}\left|Pr\left(e_{i}^{*}=e^{{*}^{\prime }}\right)-Pr\left(e_{1-i}^{*}=e^{{*}^{\prime }}\right)\right| \end{array}$$

For α is a random vector from Discrete Gaussian distribution, we can get the follow equations $Pr(e_{i}^{*}=e^{{*}^{\prime }})$ is close to $1/2^{n}$, $Pr(e_{1-i}^{*}=e^{{*}^{\prime }})$ is close to $1/2^{n}$. Therefor, we can get $\Delta (e_{i}^{*},e_{1-i}^{*})$ is close to 0.

Similarly, we can get $\Delta (y_{1}^{i},y_{1}^{1-i})$, $\Delta (y_{2}^{i},y_{2}^{1-i})$, $\Delta (\zeta_{1}^{i*},\zeta_{1}^{1-i*})$, and $\Delta (\zeta_{2}^{i*},\zeta_{2}^{1-i*})$ are close to 0. Therefore, $S^{*}$ cannot recognize *m* from $e^{*},y_{1},y_{2},\zeta_{1},\zeta_{2}$, i.e., $S^{*}$ wins the experiment with probability 1/2+η(n). Therefore, we prove the theorem.

Before proving the one-more unforgeability of IDBS-NTRU, we will define some notations as follows:

Let $\delta_{1},\delta_{2},\delta_{3},\delta_{4}$ be simulating the cost functions of *H* hash, extract oracle, $H^{{}^{\prime }}$ hash, and signature oracles respectively. Let $\eta ,\eta^{{}^{\prime }}$ be non-negligible probability, and *t* be time respectively, Θ be a polynomial time algorithm, and Γ be a polynomial time forger. ☐

**Theorem** **5**  (One-more Unforgeability)**.** *If *Γ* is able to generate a legal signature with η in t, after at most $\tau_{1},\tau_{2},\tau_{3},\tau_{4}$ times queries respectively to H hash, Extract, $H^{{}^{\prime }}$ hash, and signature oracles. Then R-$SIS_{q,1,2,\beta }^{\kappa }$ can be solved by *Θ* with probability at least $\eta^{{}^{\prime }}=\left(1-2^{-\omega (logN)}\right)\eta$ in time $t^{{}^{\prime }}=t+\tau_{1}^{\tau_{2}}\left(\tau_{1}\delta_{1}+\tau_{2}\delta_{2}\right)+$$\tau_{3}^{\tau_{4}}\left(\tau_{3}\delta_{3}+\;\tau_{4}\delta_{4}\right)$.*

**Proof.**  Assuming an adversary Γ is able to produce an IDBS signature with η, we can construct Θ, this algorithm can obtain the solution of R-SIS on the NTRU lattice. The followings are the simulated interactive environment.

ST: Θ selects $h\in R_{q}^{\times }$, $H,H^{{}^{\prime }}$ at random. Then Θ computes and sends the public parameters $paras=\{h,H,H^{{}^{\prime }},\epsilon ,q,s\}$ to the Γ.

H oracle Queries: Θ will maintains a list $L_{h}$, in the beginning, the list is mull. Once receiving an $id_{i}$, Θ will inquire $L_{h}$. If there exists a corresponding hash value $t_{i}$, Θ will return $t_{i}$. Otherwise Θ will return a random value. After that, Θ will save $id_{i},t_{i}$ in $L_{h}$.

H ${}^{\prime }$ oracle Queries: Θ maintains a list $L_{h}^{{}^{\prime }}$, in the beginning, the list is null. Once receiving $m_{i},\Lambda_{i}=y_{1_{i}}+hy_{2_{i}}+h\gamma_{i}+\alpha_{i}-\alpha_{i}H\left(id_{i}\right)$, we assume Θ has already quire H oracle and gotten an entry $id_{i},t_{i}$. Then Θ will quire $L_{h}^{{}^{\prime }}$. If there already exists a corresponding hash value $e_{i}$, Θ will return $e_{i}$. Otherwise, Θ will return a random value. After that, Θ will save $m_{i},\Lambda_{i},y_{1_{i}},y_{2_{i}},\gamma_{i},\alpha_{i},id_{i},e_{i},t_{i}$ to $L_{h}^{{}^{\prime }}$.

EX Oracle Queries: Θ maintains a list $L_{id}$, in the beginning, the list is null. Once receiving an identity $id_{i}$, Θ will inquire *H* oracle. If there does not exist a corresponding hash value in $L_{id}$, Θ will randomly selects a $t_{i}$ and return it. Otherwise, return the corresponding $t_{i}$. After that, Θ can get a $sk_{id_{i}}=\left(s_{1_{i}},s_{2_{i}}\right)$, Θ returns $sk_{id_{i}}$ to Γ as the private key related with $id_{i}$ and saves the tuple $\left(id_{i},t_{i},sk_{id_{i}}\right)$ in $L_{id}$.

SG Oracle Queries: Γ queries the signing oracle for $\left(m_{i},id_{i}\right)$. Θ checks if $id_{i}$ is already queried for *H*, $H^{{}^{\prime }}$ or extraction oracles. If it is, Θ can get an entry $\left(id_{i},t_{i},sk_{id_{i}}\right)$ from $L_{id}$. Else Θ simulates the extraction oracle and obtain a new secret key. Then Θ executes the BS protocol to obtain a valid signature $\left(m_{i},id_{i},e_{i},\zeta_{1_{i}},\zeta_{2_{i}}\right)$ and stores the value $\left(m_{i},id_{i},e_{i},\zeta_{1_{i}},\zeta_{2_{i}}\right)$ in the list $L_{S}$.

Output: Finally, Γ firstly outputs a forged signature $\left(e_{i},\zeta_{1_{i}},\zeta_{2_{i}},m_{i},id_{i}\right)$. Θ rewinds $\Gamma_{i}$ to the point where it queries $H^{{}^{\prime }}$ for $\left(m_{i},id_{i}\right)$ and obtains another signature $\left(e_{i}^{{}^{\prime }},\zeta_{1_{i}}^{{}^{\prime }},\zeta_{2_{i}}^{{}^{\prime }},m_{i},id_{i}\right)$.

Therefore, Θ can solve R-$SIS_{q,1,2,\beta }^{\kappa }$ problem over the NTRU lattice. Θ obtains $sk_{id_{i}}$ and $e_{i}^{{}^{\prime }},y_{1_{i}},y_{2_{i}},\alpha_{i},\gamma_{i}$ from the $L_{S}$. Θ computes $\zeta_{1_{i}}=y_{1_{i}}+s_{1_{i}}*e_{i}^{*}+\alpha_{i},\zeta_{2_{i}}=y_{2_{i}}+s_{2_{i}}*e_{i}^{*}+\gamma_{i}$, and $\zeta_{1_{i}}+\zeta_{2_{i}}*h-H\left(id_{i}\right)e_{i}$. Then Θ checks whether $\zeta_{1_{i}}+\zeta_{2_{i}}*h-H\left(id_{i}\right)e_{i}=\zeta_{1_{i}}^{{}^{\prime }}+\zeta_{2_{i}}^{{}^{\prime }}*h-H\left(id_{i}\right)e_{i}^{{}^{\prime }}=y_{1_{i}}+h*y_{2_{i}}+h\gamma_{i}+\alpha_{i}-\alpha_{i}H\left(id_{i}\right)$. If they are not equal, there is a collision of $H^{{}^{\prime }}$. If $\left(\zeta_{1_{i}},\zeta_{2_{i}}\right)\neq \left(\zeta_{1_{i}}^{{}^{\prime }},z_{2_{i}}^{{}^{\prime }}\right)$, we can get $\left(\zeta_{1_{i}}-\zeta_{1_{i}}^{{}^{\prime }}\right)+h\left(\zeta_{2_{i}}-\zeta_{2_{i}}^{{}^{\prime }}\right)=0$ and $\left|\right|\left(\zeta_{1_{i}}-\zeta_{1_{i}}^{{}^{\prime }},\zeta_{2_{i}}-\zeta_{2_{i}}^{{}^{\prime }}\right)\left|\right|\leq 8s\sqrt{2N}$. So $\left(\zeta_{1_{i}}-\zeta_{1_{i}}^{{}^{\prime }},\zeta_{2_{i}}-\zeta_{2_{i}}^{{}^{\prime }}\right)$ is one solution to R-$SIS_{q,1,2,\beta }^{\kappa }$.

Now we start to analyze the advantage of Θ. As discussed above, Θ wins the game if and only if Γ has successfully forged $\left(\zeta_{1}^{{}^{\prime }},\zeta_{2}^{{}^{\prime }},u^{{}^{\prime }}\right)$ and $\left(\zeta_{1},\zeta_{2}\right)\neq \left(\zeta_{1}^{{}^{\prime }},\zeta_{2}^{{}^{\prime }}\right)$. Next according to the Lemma 4.6 in [34], Γ can solve R-$SIS_{q,1,2,\beta }^{\kappa }$ with probability at least $\eta^{{}^{\prime }}=\left(1-2^{-\omega (logN)}\right)\eta$, where $\beta =8s\sqrt{2N}$. It is obviously that $t^{{}^{\prime }}=t+\tau_{1}^{\tau_{2}}\left(\tau_{1}\delta_{1}+\tau_{2}\delta_{2}\right)+\tau_{3}^{\tau_{4}}\left(\tau_{3}\delta_{3}+\tau_{4}\delta_{4}\right)$. We prove this theorem. ☐

### 4.2. Performances

Here, we will compare our IDBS-NTRU’s performances with other IDBS schemes. First of all, we will compare NTRU-IDBS scheme with traditional IDBS schemes in terms of performance, which were constructed based on number theory. Secondly, we will compare our IDBS-NTRU scheme with lattice-based BS schemes in terms of performance. 

(1) Comparing with traditional IDBS schemes 

As shown in Table 1, we compare IDBS-NTRU’performance with ZK scheme [35], HCZ scheme [10], and CZYW scheme [36]. The ZK scheme is constructed based on computational diffie-hellman problem of bilinear pairings. The HCZ scheme is constructed based on discrete logarithm problem of ellipse curve. The CZYW scheme is constructed based on big integer factoring problem. The IDBS-NTRU scheme’s signing speed and verification speed are O(n), which outperform ZK scheme, HCZ scheme, and CZYW schemes. Its moves are 2, it is shorter than ZK scheme and HCZ scheme. Its signing secret key is $2nlog(s\sqrt{n})$, it is larger than ZK scheme and HCZ scheme. However, the rsa has to use $O(n^{3})$ to achieve n bits security, the signing secret key of IDBS-NTRU scheme is shorter than CZYW scheme. The signature size of IDBS-NTRU scheme is 2nlog(12σ)+n(logλ+1), it is larger than ZK, HCZ, and CZYW schemes. For the same reason, it is also shorter than CZYW scheme. The most important of all, the BS schemes based on number theory are considered to be insecure to resist quantum computers attack [4], our IDBS-NTRU scheme is more secure than other three traditional schemes.

(2) Comparing with lattice-based BS schemes 

We compare IDBS-NTRU’s performance with GHWX [37], ZTZ [4], Rückert [32], and ZM schemes [38] in Table 2, *n* denotes safety parameter. GHWX scheme and ZM scheme are constructed based on small integer solution problem of lattice. ZTZ scheme is constructed based on closest vector problem of lattice. Rückert is constructed based on ideal-lattice shortest vector problem.

As presented in Table 2, IDBS-NTRU’s signing speed is O(n), which outperforms all the other schemes. IDBS-NTRU’s verification speed is O(n), which outperforms GHWX and ZM schemes. Our IDBS-NTRU scheme has two moves, it is shorter than Rückert scheme, and ZM scheme. In Rückert scheme, the parameters satisfy $m>\lfloor c_{m}log\left(1\right)\rfloor +1$, $c_{m}>1/log\left(2d_{s}\right)$. In ZM schemes, the parameters satisfy m>2nlogq,q>2. The signing secret key of our IDBS-NTRU scheme is $2nlog(s\sqrt{n})$, it is shorter than all the other schemes. The signature size of our IDBS-NTRU scheme is 2nlog(12σ)+n(logλ+1), it is shorter than Rückert scheme, but it is larger than GHWX, ZTZ, and ZM schemes. The ZTZ scheme and Rückert scheme are not identity-based scheme, they depend on the public key infrastructure. However, our IDBS-NTRU scheme does not need to dependent on public key infrastructure.

## 5. Conclusions

In this work, we present an IDBS-NTRU scheme by using NTRU lattice, this scheme can protect user privacy and guarantee the trustworthy of big data in e-payment and e-voting systems in wireless sensor networks, this scheme has the advantages of NTRU Lattice such as high efficiency, compact key, high security after appropriate parameterized etc. Our scheme is secure and efficient. Furthermore, we prove IDBS-NTRU satisfies blindness and unforgeability. In addition, comparing with traditional IDBS schemes, IDBS-NTRU outperforms other IDBS schemes in terms of signing speed and verifying speed. Comparing with lattice-based schemes, IDBS-NTRU scheme outperforms other schemes in terms of signing speed, verifying speed, and signing secret key, outperforms Rückert scheme in terms of signature size moves and signature size, and outperforms ZM scheme in terms of moves. The schemes based on number theorem are considered insecure to resist the quantum computers attack, so our scheme is more secure than them. Furthermore, lattice-based schemes usually have a lot of parameters which need to be initialized correctly, these schemes are not easy to implement. Therefore, almost all the works related with lattice-based cryptography are still in the step of theory research.

In addition, if we can add some common message such as date between the signer and a user in our scheme, it is easy to transform our scheme into an identity-based partial BS scheme, which is suitable for the real e-payment and e-voting systems. In the future, we will continue to construct a partial IDBS scheme based on lattice.

## Figures and Tables

**Table 1:** Performance comparison with traditional IDBS schemes.

IDBS Scheme	ZK [35]	HCZ [10]	CZYW [36]	Ours
Hard Problem	CDHP	DLP	Factoring	R-SIS
Signing Speed	$O(n^{3})$	$O(n^{3})$	$O(n^{3})$	O(n)
Verifying Speed	$O(n^{3})$	$O(n^{3})$	$O(n^{3})$	O(n)
Moves	3	3	2	2
Signing Secret key	2*n*	logk+2*n*	*n*	$2nlog(s\sqrt{n})$
Signature size	3*n*	logk+4*n*	*n*	2nlog(12σ)+n(logλ+1)

**Table 2:** Performance Comparison with lattice-based BS schemes.

Lattice-Based BS Scheme	GHWX [37]	ZTZ [4]	Rückert [32]	ZM [38]	Ours
Hard Problem	SIS	CVP	ISVP	SIS	R-SIS
Signing Speed	$O(n^{2})$	$O(n^{2}logn)$	$O(n{\left(logn\right)}^{c})$	$O(n^{2})$	O(n)
Verifying Speed	$O(n^{3})$	O(*n*)	O(*n*)	$O(n^{3})$	O(*n*)
Moves	2	2	4	3	2
Signing secret key	nmlog(q+1)	$dn^{2}\left(\log n+1\right)$	$mn\log\left(2d_{s}+1\right)$	$m^{2}\log\left(q+1\right)$	2n $log(s\sqrt{n})$
Signature size	mlog(q+1)	dn(logn+1)	$n^{2}+mn$ $\log\left(2mnd_{s}d_{\epsilon^{*}}\right)$	2mlog(q+1)	2nlog(12σ)+ n(logλ+1)
Identity Based	yes	no	no	yes	yes

## References

[1] Ahmad S., Hang L., Kim D.H. (2018). Design and Implementation of Cloud-Centric Configuration Repository for DIY IoT Applications. Sensors.

[2] Gaur A., Scotney B., Parr G., Mcclean S. (2015). Smart City Architecture and its Applications Based on IoT. Procedia Comput. Sci..

[3] Guan Z., Li J., Wu L., Zhang Y., Wu J., Du X. (2017). Achieving Efficient and Secure Data Acquisition for Cloud-Supported Internet of Things in Smart Grid. IEEE Internet Things J..

[4] Zhu H.F., Tan Y.A., Zhang X.S., Zhu L.H., Zhang C.Y., Zheng J. (2017). A round-optimal lattice-based blind signature scheme for cloud services. Future Gener. Comput. Syst..

[5] Zhang X., Tan Y.A., Chen L., Yuanzhang L., Ji L. (2018). A Covert Channel over VoLTE via Adjusting Silence Periods. IEEE Access.

[6] Gao C.Z., Cheng Q., He P., Susilo W., Li J. (2018). Privacy-Preserving Naive Bayes Classifiers Secure against the Substitution-then-Comparison Attack. Inf. Sci..

[7] Li P., Li T., Ye H., Li J., Chen X., Xiang Y. (2018). Privacy-preserving machine learning with multiple data providers. Future Gener. Comput. Syst..

[8] Guan Z., Si Z.X., Wu L., Guizani N., Du X., Ma Y. (2018). Privacy-preserving and Efficient Aggregation based on Blockchain for Power Grid Communications in Smart Communities. IEEE Internet Things J..

[9] Zheng J., Tan Y.A., Zhang Q., Zhang X., Zhu L., Zhang Q. (2018). Cross-cluster asymmetric group key agreement for wireless sensor networks. Sci. China Inf. Sci..

[10] He D., Chen J., Zhang R. (2011). An efficient identity-based blind signature scheme without bilinear pairings. Comput. Electr. Eng..

[11] Peikert C. (2016). A Decade of Lattice Cryptography.

[12] Wang Z., Chen X., Wang P. (2017). Adaptive-ID Secure Identity-Based Signature Scheme from Lattices in the Standard Model. IEEE Access.

[13] Xie J., Hu Y.P., Gao J.T., Gao W. (2016). Efficient identity-based signature over NTRU lattice. Front. Inf. Technol. Electron. Eng..

[14] Lyubashevsky V., Pointcheval D., Johansson T. (2012). Lattice Signatures without Trapdoors. Advances in Cryptology—EUROCRYPT 2012.

[15] Zhu H.F., Tan Y.A., Yu X., Xue Y., Zhang Q.X., Zhu L.H., Li Y.Z. (2018). An Identity-Based Proxy Signature on NTRU Lattice. Chin. J. Electron..

[16] Zhang X.S., Liang C., Zhang Q.X., Li Y.Z., Zheng J., Tan Y.A. (2018). Building covert timing channels by packet rearrangement over mobile networks. Inf. Sci..

[17] Xue Y., Tan Y.A., Liang C., Li Y., Zheng J., Zhang Q. (2018). RootAgency: A digital signature-based root privilege management agency for cloud terminal devices. Inf. Sci..

[18] Tan Y.A., Xue Y., Liang C., Zheng J., Zhang Q.X., Zheng J., Li Y.Z. (2018). A root privilege management scheme with revocable authorization for Android devices. J. Netw. Comput. Appl..

[19] Lin Q., Li J., Huang Z., Chen W., Shen J. (2018). A short linearly homomorphic proxy signature scheme. IEEE Access.

[20] Lin Q., Yan H., Huang Z., Chen W., Shen J., Tang Y. (2018). An ID-based linearly homomorphic signature scheme and its application in blockchain. IEEE Access.

[21] Xu J., Wei L., Zhang Y., Wang A., Zhou F., Gao C. (2018). Dynamic Fully Homomorphic encryption-based Merkle Tree for lightweight streaming authenticated data structures. J. Netw. Comput. Appl..

[22] Yu X., Zhang C., Xue Y., Zhu H., Li Y., Tan Y.A. (2018). An extra-parity energy saving data layout for video surveillance. Multimed. Tools Appl..

[23] Liu Z., Huang Y., Li J., Cheng X., Shen C. (2018). DivORAM: Towards a Practical Oblivious RAM with Variable Block Size. Inf. Sci..

[24] Li T., Li J., Liu Z., Li P., Jia C. (2018). Differentially Private Naive Bayes Learning over Multiple Data Sources. Inf. Sci..

[25] Yu X., Tan Y.A., Zhang C., Liang C., Aourra K., Zheng J., Zhang Q. (2018). A High-Performance Hierarchical Snapshot Scheme for Hybrid Storage Systems. Chin. J. Electron..

[26] Li J., Sun L., Yan Q., Li Z., Srisa-an W., Ye H. (2018). Significant Permission Identification for Machine Learning Based Android Malware Detection. IEEE Trans. Ind. Inform..

[27] Shen J., Gui Z., Ji S., Shen J., Tan H., Tang Y. (2018). Cloud-aided lightweight certificateless authentication protocol with anonymity for wireless body area networks. J. Netw. Comput. Appl..

[28] Xue Y., Tan Y.A., Liang C., Zhang C., Zheng J. (2017). An optimized data hiding scheme for Deflate codes. Soft Comput..

[29] Gentry C., Peikert C., Vaikuntanathan V. (2008). Trapdoors for Hard Lattices and New Cryptographic Constructions. Proceedings of the Fortieth Annual ACM Symposium on Theory of Computing—STOC 2008.

[30] Schröder D., Unruh D. (2017). Security of Blind Signatures Revisited. J. Cryptol..

[31] Zhu H.F., Tan Y.A., Zhu L.H., Zhang Q.X., Li Y.Z. (2018). An Efficient Identity-Based Proxy Blind Signature for Semioffline Services. Wirel. Commun. Mob. Comput..

[32] Rückert M., Abe M. (2010). Lattice-Based Blind Signatures. Advances in Cryptology—ASIACRYPT 2010.

[33] Boneh D., Kim S., Nikolaenko V., Gollmann D., Miyaji A., Kikuchi H. (2017). Lattice-Based DAPS and Generalizations: Self-enforcement in Signature Schemes. Applied Cryptography and Network Security, Proceedings of the 15th International Conference, ACNS 2017, Kanazawa, Japan, 10–12 July 2017.

[34] Güneysu T., Lyubashevsky V., Pöppelmann T. (2015). Lattice-based signatures: optimization and implementation on reconfigurable hardware. IEEE Trans. Comput..

[35] Zhang F., Kim K. (2002). ID-based blind signature and ring signature from pairings. Advances in Cryptology—ASIACRYPT 2002.

[36] Cheng X., Zhu H., Yang C., Wang X. (2005). Identity-based Blind and Verifiably Encrypted Signatures from RSA. Information Security and Cryptology.

[37] Gao W., Hu Y., Wang B., Xie J., Chen K., Lin D., Yung M. (2017). Identity-Based Blind Signature from Lattices in Standard Model. Information Security and Cryptology.

[38] Zhang L., Ma Y. (2014). A lattice-based identity-based proxy blind signature scheme in the standard model. Math. Probl. Eng..

